# Glucagon-like peptide-1 receptor agonists and aesthetic surgery: perioperative safety, timing, durability and body composition

**DOI:** 10.3389/fendo.2026.1969433

**Published:** 2026-10-05

**Authors:** Richa Chaturvedi, Kuldeep Singh, Rameshwari R. Chakole, Bhupesh K. Yadav, Juli Dhameliya, Deepali Joshi

**Affiliations:** 1 Advance Center for Obesity, Diabetes and Endocrinology (ACODE), Indraprastha Apollo Hospital, New Delhi, India; 2 Department of Aesthetic, Plastic and Reconstructive Surgery, Indraprastha Apollo Hospital, New Delhi, India

**Keywords:** aesthetic surgery, body contouring, GLP-1 receptor agonists, lean mass, obesity pharmacotherapy, perioperative management, sarcopenic obesity, weight regain

## Abstract

Glucagon-like peptide-1 receptor agonists (GLP-1 RAs) and dual incretin agonists have transformed the management of obesity and type 2 diabetes and are rapidly reshaping the population seeking aesthetic surgery. By producing double-digit percentage weight loss, these agents expand eligibility for elective procedures while generating new patterns of skin redundancy, soft-tissue laxity, and facial volume depletion that drive demand for body contouring and facial rejuvenation. At the same time, incretin therapy introduces distinct perioperative considerations: delayed gastric emptying with possible pulmonary aspiration in symptomatic patients, gastrointestinal adverse effects that peak during dose escalation, loss of lean mass with rapid weight reduction, nutritional inadequacy during profound appetite suppression, and frequent weight regain after drug discontinuation, which threatens the durability of surgical results. This mini-review synthesizes current evidence on the mechanisms of GLP-1 RAs relevant to the reconstructive surgical patient, summarizes the evolving guidance from anesthesia societies, moving from blanket preoperative withholding to individualized risk stratification, reviews emerging outcome data on body contouring in GLP-1 RA users, and discusses body composition, sarcopenia risk, and nutritional optimization. We propose a pragmatic framework, anchored in weight stability and symptom burden, for timing elective aesthetic procedures relative to GLP-1 RA initiation, escalation, and maintenance, along with a multidisciplinary perioperative algorithm that links the endocrinologist, plastic surgeon, and anesthesiologist. As incretin-based pharmacotherapy expands globally, coordinated endocrine–surgical–anesthetic care will be essential to maximize safety, functional recovery, and patient satisfaction in the GLP-1 era.

## 1 Introduction

Anti-obesity pharmacotherapy has entered routine clinical practice. In pivotal phase 3 trials, once-weekly semaglutide 2.4 mg produced a mean weight reduction of 14.9% at 68 weeks (1), and the dual glucose-dependent insulinotropic polypeptide (GIP)/glucagon-like peptide-1 (GLP-1) receptor agonist tirzepatide achieved up to 20.9% (2). Uptake has been extraordinary: by 2024, approximately one in eight adults in the United States reported ever having used a GLP-1 receptor agonist (GLP-1 RA) (3). The aesthetic surgery population has shifted in parallel. In its 2024 procedural statistics, the American Society of Plastic Surgeons (ASPS) reported, for the first time, on weight-loss medication use, identifying more than 800,000 aesthetic patients on these drugs; among GLP-1 RA users under member care, 20% had already undergone plastic surgery, and a further 39% were considering a surgical procedure (4).

Historically, substantial weight loss was achieved primarily through bariatric surgery and was managed by plastic surgeons through structured pathways that incorporated nutritional surveillance, supplementation, and staged body contouring (5). In contrast, pharmacotherapy-associated weight loss affects a much broader population, often without endocrinology follow-up, dietetic support, or formal preoperative assessment, and can progress rapidly during dose escalation. This creates a novel clinical scenario in which plastic surgeons, anesthesiologists, and endocrinologists must jointly manage dynamic weight trajectories, altered body composition, gastrointestinal adverse effects, and long-term weight maintenance.

This mini review synthesizes evidence from searches of PubMed, Embase, and Google Scholar through August 2026, combining terms for GLP-1 RAs and dual/triple agonists with terms for aesthetic surgery, body contouring, perioperative management, gastric emptying, anesthesia, body composition, and weight regain, prioritizing randomized trials, large cohort studies, meta-analyses, and society guidance. We focus on four clinically actionable domains: perioperative safety, procedural timing, body composition and nutrition, and durability of aesthetic outcomes.

## 2 Mechanisms of incretin-based therapy, relevant to the surgical patient

GLP-1 RAs reduce energy intake predominantly through central appetite suppression and enhanced satiety, with additional slowing of gastric emptying mediated by vagal pathways (6). Deceleration of gastric emptying is most pronounced after initiation and dose escalation and is subject to partial tachyphylaxis with continued exposure to long-acting agents, although substantial interindividual variability persists. Nausea, vomiting, reflux, and constipation are the most common adverse effects and cluster during the escalation phase (7). These gastrointestinal effects, rather than weight loss itself, underpin the principal anaesthetic concern: residual gastric content despite guideline-concordant fasting.

Metabolic effects are largely favorable for surgery. In patients with type 2 diabetes, improved glycemic control attenuates the immune dysfunction, microvascular compromise, and collagen abnormalities associated with hyperglycemia (8), and diabetes itself is an established risk factor for surgical site infection (9). Weight reduction also improves obstructive sleep apnea, ventilation, and positioning - factors that have historically limited elective surgery in patients with obesity. The countervailing concern is nutritional: profound appetite suppression can reduce protein and micronutrient intake below requirements during the period when patients seek surgery, with implications for wound healing, immune competence, and lean mass preservation (10).

## 3 The changing aesthetic surgery population

Medication-associated weight loss is increasingly followed by presentations of excess skin, soft-tissue laxity, and deflation-type facial ageing, driving demand for abdominoplasty, brachioplasty, thigh lift, mastopexy, and facial rejuvenation. ASPS data show continued growth in skin-tightening procedures, with 171,064 abdominoplasties (+1%), arm lifts (+2%), and buttock/thigh lifts (+3%), against a stable overall cosmetic surgery volume of approximately 1.6 million procedures (4). Facial changes attract particular attention. Rapid depletion of the malar, buccal, and temporal fat compartments produces the gaunt, prematurely aged appearance colloquially termed “Ozempic face”. A 2025 systematic review of 23 studies documented preferential facial adipose loss without compensatory collagen remodeling and showed that public search interest in GLP-1 facial changes tracks rising interest in fillers, fat grafting, and facelift surgery (11).

The GLP-1 phenotype differs from the classic post-bariatric phenotype in clinically relevant ways: total weight loss is usually smaller but still substantial; the trajectory may not have a clearly defined endpoint; pharmacotherapy frequently continues indefinitely (or is stopped and restarted); and patients typically lack the structured nutritional follow-up of bariatric pathways (5). Surgeons should also anticipate pressure from patients eager to consolidate their transformation while still actively losing weight, a scenario that predisposes to unpredictable contouring and revision surgery. Expectation-setting should acknowledge that skin quality after pharmacologic weight loss varies with age, sun exposure, rate of weight loss, and magnitude of loss, and that nonsurgical skin-tightening modalities have limited efficacy for true redundancy.

## 4 Perioperative safety: gastric emptying, aspiration risk and evolving anesthesia guidance

Concerns about periprocedural aspiration arose from converging observational data. In a retrospective analysis of elective endoscopy, semaglutide use was associated with increased residual gastric content (24.2% vs 5.1%) despite adequate fasting (12). Gastric ultrasound studies corroborated this: among fasted volunteers on semaglutide, residual solids were significantly more prevalent than in controls (13). In a cross-sectional study of elective surgical patients, 56% of GLP-1 RA users had increased residual gastric content on point-of-care ultrasound versus 19% of non-users, despite standard fasting (14). Population-scale data suggest the clinical correlate is real but modest: among nearly one million endoscopy patients, GLP-1 RA use was associated with a hazard ratio of 1.33 for aspiration pneumonia (15). Reassuringly, in 23,679 patients with diabetes undergoing emergency surgery, where withholding was not possible, preoperative GLP-1 RA use was not associated with increased postoperative respiratory complications (4.0% vs 3.9%) (16).

Guidance has evolved accordingly. The 2023 American Society of Anesthesiologists (ASA) consensus advised holding daily agents on the day of elective procedures and weekly agents for one week before (17, 18), an approach criticized for disrupting glycemic control and for lacking outcome evidence. The 2024 multisociety guidance (ASA, American Gastroenterological Association, American Society for Metabolic and Bariatric Surgery, International Society of Perioperative Care of Patients with Obesity, and Society of American Gastrointestinal and Endoscopic Surgeons) moved decisively towards individualized risk assessment: most patients may continue therapy perioperatively with risk mitigation; a 24-hour liquid diet before the procedure, symptom screening, point-of-care gastric ultrasound where available, and rapid-sequence induction or deferral when significant gastrointestinal symptoms or retained content are identified (19). The 2025 Society for Perioperative Assessment and Quality Improvement (SPAQI) consensus (20), Australasian multi-college recommendations (21), and gastroenterology rapid updates (22) adopt broadly concordant, escalation-phase- and symptom-based algorithms.

In aesthetic practice, several points merit emphasis. First, elective cosmetic surgery is by definition deferrable, so unresolved gastrointestinal symptoms, active dose escalation, or uncertain fasting status should prompt postponement rather than mitigation. Second, many aesthetic procedures can be performed under local anesthesia or minimal sedation with preserved airway reflexes, which carries a lower aspiration risk than deep sedation or general anesthesia; the depth of planned sedation, rather than the procedure itself, should anchor risk stratification. Third, decisions to withhold therapy should involve the prescribing clinician: abrupt discontinuation may destabilize glycaemia in diabetes, requiring a bridging plan for patients for whom the GLP-1 RA is the principal glucose-lowering agent, and, if prolonged, may trigger rebound appetite and weight regain that itself compromises the surgical plan. Long half-lives also mean that holding a weekly agent for a single dose does not guarantee normalization of gastric emptying, further supporting symptom-based rather than purely schedule-based strategies (7, 19).

Postoperative care should anticipate the same physiology. Multimodal antiemetic prophylaxis is prudent given the baseline susceptibility to nausea, and early oral intake may be limited. Hydration, antiemetic cover, and a graduated diet with explicit protein targets help prevent catabolic stress and unplanned admission. Society guidance supports resuming or continuing incretin therapy once a regular diet is tolerated, in coordination with the prescriber (19, 20).

## 5 Timing of elective aesthetic procedures

No prospective evidence defines the optimal interval between pharmacotherapy-induced weight loss and body contouring; principles are extrapolated from the post-bariatric literature, where surgery is deferred until a stable plateau, typically 12–18 months after surgery (5). Applied to GLP-1 RAs, a pragmatic framework aligns major excisional contouring with (i) weight stability - commonly operationalized as less than 2–3 kg fluctuation over at least three months on a stable maintenance dose, (ii) resolution or control of gastrointestinal symptoms outside the escalation phase, (iii) documented nutritional adequacy, particularly protein intake, and (iv) glycemic stability in diabetes, given the association of perioperative hyperglycemia with infection and impaired healing (9, 23). Operating during active weight loss risks residual laxity, contour irregularity, and early revision. Facial volume restoration may be staged reasonably but should be counselled with the possibility of further deflation if weight loss continues. When multiple regions require correction, sequencing follows post-bariatric principles, prioritizing the anatomic unit of greatest functional and psychological burden (most often the abdominal pannus), separating major excisional stages, and re-verifying weight stability before each stage (5).

Whether to pause therapy before surgery for wound-healing reasons, as distinct from anesthetic reasons, remains unresolved. A retrospective comparative study of 80 lipoabdominoplasty patients reported complication rates of 45% when semaglutide was continued until surgery, 30% with a two-week pause, and 10% with a four-week pause, the latter matching semaglutide-naïve controls (24). These provocative data await replication and conflict with larger cohorts showing no overall excess risk (Section 7); at present, they support individualized, consensus-based decisions rather than mandatory discontinuation.

## 6 Body composition: fat mass, lean mass and nutritional optimization

Weight loss by any means includes fat-free mass, typically about one-quarter of the total (25). In the STEP 1 dual-energy X-ray absorptiometry (DXA) substudy, lean mass accounted for approximately 40% of total mass lost with semaglutide, although the lean-to-total body mass ratio improved (1). In the SURMOUNT-1 substudy, roughly 75% of tirzepatide-induced weight loss was fat mass and 25% lean mass, with no disproportionate lean loss in older participants and improved muscle composition (reduced intermuscular fat) (26). The functional significance of pharmacotherapy-associated lean mass loss is debated, but consensus is emerging that muscle mass, strength, and function should be actively protected during medically induced weight loss, particularly in older adults, in whom sarcopenia may be unmasked or accelerated (27, 28).

For the surgical candidate, these changes matter twice over. Physiologically, protein-energy inadequacy impairs collagen deposition and wound tensile strength, and slows functional recovery (8, 29). Aesthetically, loss of subcutaneous fat reduces structural support for redundant skin, and depletion of facial fat pads drives demand for volume restoration (11). Preoperative optimization should therefore include: protein intake of approximately 1.2–1.6 g/kg/day during active weight loss and the perioperative period; structured resistance training to attenuate lean mass loss during caloric restriction (29–31); screening for micronutrient deficiencies (iron, B12, vitamin D) in patients with prolonged appetite suppression; and sarcopenia risk assessment (e.g., SARC-F, grip strength, gait speed) in older adults (10, 28). Pharmacologic muscle preservation is an active frontier: in a phase 2 trial, adding the activin type II receptor antibody bimagrumab to semaglutide increased the proportion of weight lost as fat to over 92% while largely preserving lean mass (32). Analogous agents are in late-phase development.

## 7 Outcomes of aesthetic surgery in GLP-1 RA users

Direct outcome data, although early and largely retrospective, are broadly reassuring. In a 10-year, single-institution, propensity-matched analysis of 590 body-contouring patients, 30-day readmission, dehiscence, seroma, and hematoma rates did not differ between GLP-1 RA users and controls (33). A prospective comparison of lipoabdominoplasty patients on semaglutide found no increase in major complications, with more frequent minor bruising and hyperpigmentation (34). A 2026 systematic review and meta-analysis pooling data from 8,944 patients found no significant differences in surgical site infection, seroma, hematoma, or composite surgical site occurrences; a borderline increase in wound dehiscence (5.2% vs 2.9%; risk ratio 1.62) was driven by a single large cohort and lost significance in sensitivity analysis (35). Set against the discontinuation-timing signal described above (24), the aggregate evidence supports proceeding with surgery in optimized, weight-stable GLP-1 RA users. This reassurance should nonetheless be qualified during counselling, because the wound-healing evidence base remains preliminary in four specific respects. First, the available studies are observational and predominantly single-center, and none was designed or powered with wound healing as a primary endpoint. Second, exposure is inconsistently characterized; agent, dose, cumulative duration, and whether therapy was continued or withheld before surgery are frequently unreported, so comparisons are made between heterogeneous “users” and non-users rather than between defined perioperative strategies. Third, wound-outcome definitions are not standardized, and follow-up is typically limited to 30 days. Fourth, the plausible mediators of impaired healing in this population- rate of weight loss, protein-energy adequacy and lean mass trajectory- are seldom recorded, so residual confounding cannot be excluded. Patients should therefore be counselled that current data show no signal of harm but do not establish equivalence.

## 8 Durability, weight regain and counselling

Discontinuation of incretin therapy is usually followed by substantial regain: participants regained two-thirds of lost weight within one year of stopping semaglutide (36); in STEP 4, withdrawal after 20 weeks led to progressive regain compared with continued treatment (37); and in SURMOUNT-4, stopping tirzepatide produced a 14% regain over 52 weeks, versus a further 5.5% loss with continuation (38). Real-world persistence is poor, with nearly two-thirds of adults without diabetes discontinuing within one year, although many reinitiate (39). Because weight regain after excisional contouring degrades results and increases revision risk, preoperative counselling must explicitly address the maintenance strategy: intention to continue pharmacotherapy (with nutritional and exercise co-therapy), the risk of regain if therapy stops, realistic expectations, and staged procedures where indicated. Conversely, for patients who continue therapy, surgeons should recognize that ongoing modest weight changes and persistent appetite suppression may subtly alter contour and nutrition during the healing phase. This conversation is inherently multidisciplinary and is best co-owned by the prescribing endocrinologist, who can align maintenance dosing, nutritional co-therapy, and follow-up with the surgical calendar.

## 9 Discussion and future direction

The GLP-1 era is redefining the boundaries of aesthetic surgery. The same drugs that render millions newly eligible for elective procedures impose a distinctive perioperative signature: residual gastric content and a modest but real aspiration signal in symptomatic patients; nutritional and lean-mass vulnerability during rapid weight loss; and fragile durability tethered to long-term drug persistence. **Table 1** summarizes benefits, concerns, and practical actions across these domains, and **Figure 1** presents a multidisciplinary algorithm for timing and perioperative planning that operationalizes current society guidance (19, 20) around a simple axis - weight trajectory and symptom burden, with elective surgery reserved for the weight-stable, symptom-controlled, nutritionally replete patient.

**Table 1:** GLP-1 receptor agonists and aesthetic surgery: potential benefits, concerns, and practical actions.

Domain	Potential benefit	Potential concern	Practical action
Metabolic health	Improved glycemia and cardiometabolic risk profile (1, 2, 23)	Surgery undertaken before metabolic stability is reached	Verify HbA1c and weight trajectory; optimize comorbidities before scheduling
Procedure demand	Expanded eligibility for elective aesthetic surgery (4)	Skin redundancy, soft-tissue laxity, facial volume depletion (11)	Counsel on staged procedures and realistic expectations
Anaesthetic safety	Emergency-surgery cohort data reassuring (16)	Residual gastric content; aspiration risk in symptomatic patients (12–15)	GI symptom screen; 24-h liquid diet; gastric POCUS; rapid-sequence induction or deferral (19, 20)
Timing	Predictable excision planning at weight plateau	Unpredictable contour and revision risk during active weight loss	Defer major contouring until ≥3 months of weight stability on maintenance dose (5)
Body composition	Substantial fat mass reduction; improved muscle composition (26)	Lean mass loss and sarcopenia risk, especially in older adults (1, 25, 27)	Protein 1.2–1.6 g/kg/day; resistance training; sarcopenia screening (10, 28–30)
Wound healing and outcomes	Matched-cohort complication rates comparable to non-users (33–35)	Possible dehiscence signal; optimal discontinuation timing unresolved (24, 35)	Optimize nutrition and glycemia; individualized continue-vs-hold decision with the prescriber
Durability of results	Maintenance therapy supports stable aesthetic results (37, 38)	Weight regain after discontinuation; poor real-world persistence (36, 39)	Explicit long-term weight-maintenance plan and follow-up agreed upon before surgery

GI, gastrointestinal; HbA1c, glycated hemoglobin; POCUS, point-of-care ultrasound. Numbers in parentheses refer to the reference list.

**Figure 1:**
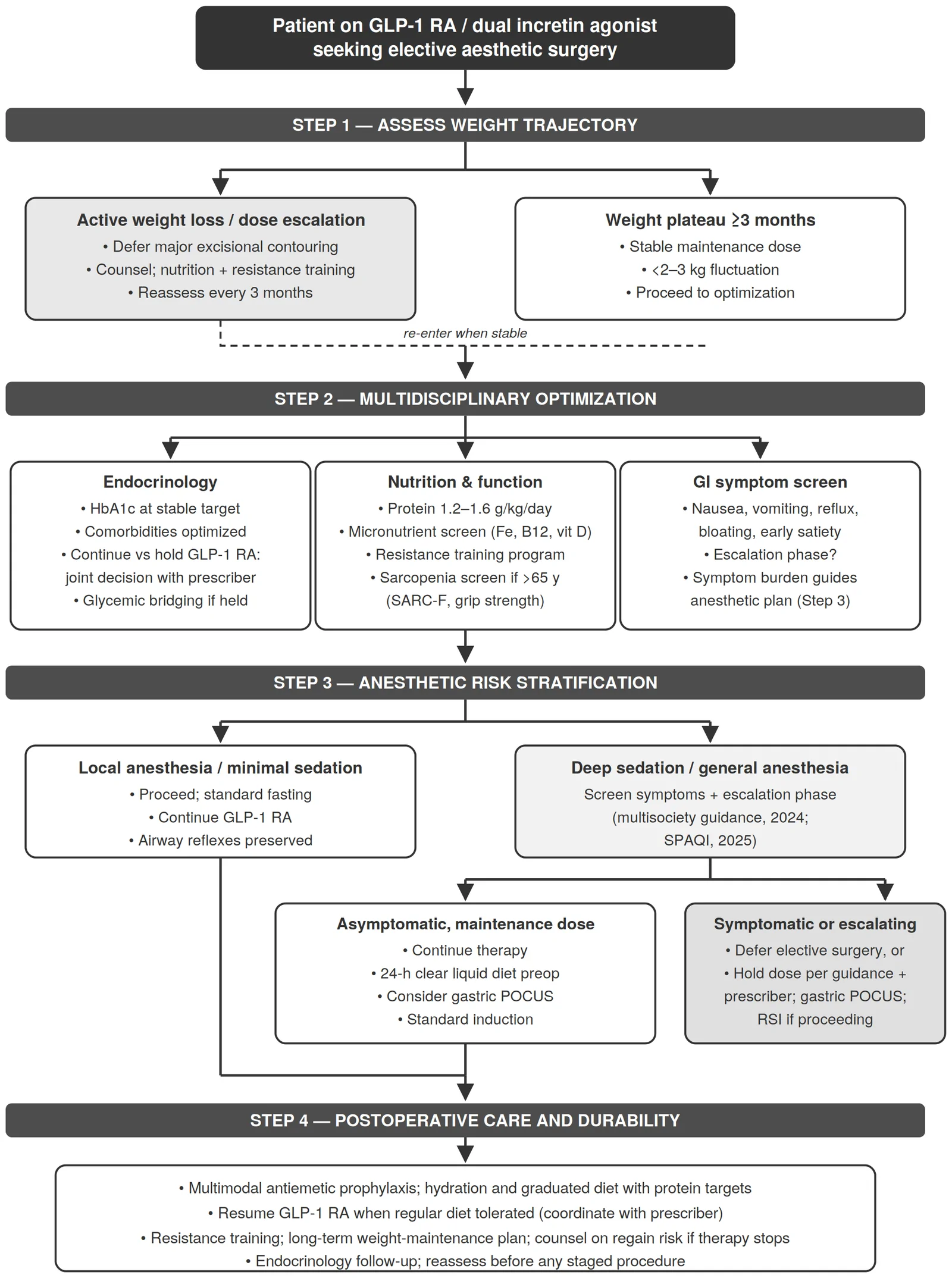
Practical multidisciplinary algorithm for timing and perioperative planning in patients using GLP-1 receptor agonists who are seeking elective aesthetic surgery. Major excisional body contouring is aligned with a sustained weight plateau; anesthetic risk stratification follows current multisociety (2024) and SPAQI (2025) guidance, anchored to gastrointestinal symptom burden and planned depth of sedation rather than to medication schedule alone. GLP-1 RA, glucagon-like peptide-1 receptor agonist; GI, gastrointestinal; HbA1c, glycated hemoglobin; POCUS, point-of-care gastric ultrasound; RSI, rapid-sequence induction; SARC-F, strength, assistance with walking, rising from a chair, climbing stairs, and falls questionnaire; SPAQI, Society for Perioperative Assessment and Quality Improvement.

Key evidence gaps persist. Prospective studies should define optimal timing windows after pharmacotherapy-associated weight loss; quantify aspiration risk in aesthetic surgical populations under modern mitigation protocols; determine whether brief preoperative discontinuation improves wound healing or merely destabilizes weight and glycemia; characterize body composition trajectories and sarcopenia risk in surgical candidates; and test protein and resistance training prescriptions powered for surgical outcomes. These questions will intensify as more potent agents, such as triple agonists like retatrutide (40) and amylin-based combinations (41), deliver near-bariatric weight loss pharmacologically, and as muscle-preserving co-therapies reach the clinic (32).

In conclusion, GLP-1 RAs are reshaping aesthetic surgery by expanding eligibility, altering tissue quality, and introducing new perioperative physiology. Elective procedures are best timed after weight stabilization, with symptom-based anesthetic risk stratification, deliberate support for protein intake and resistance training, and explicit weight-maintenance planning. A coordinated framework linking endocrinology, plastic surgery, and anesthesiology translates a rapidly evolving pharmacologic landscape into safe, durable, and satisfying surgical outcomes.
